# Baseline Performance Predicts tDCS-Mediated Improvements in Language Symptoms in Primary Progressive Aphasia

**DOI:** 10.3389/fnhum.2017.00347

**Published:** 2017-06-30

**Authors:** Eric M. McConathey, Nicole C. White, Felix Gervits, Sherry Ash, H. Branch Coslett, Murray Grossman, Roy H. Hamilton

**Affiliations:** ^1^Laboratory for Cognition and Neural Stimulation, Department of Neurology, University of PennsylvaniaPhiladelphia, PA, United States; ^2^Penn Frontotemporal Degeneration CenterPhiladelphia, PA, United States; ^3^Neurology, Perelman School of MedicinePhiladelphia, PA, United States

**Keywords:** primary progressive aphasia, tDCS, non-invasive brain stimulation, language therapy

## Abstract

Primary Progressive Aphasia (PPA) is a neurodegenerative condition characterized by insidious irreversible loss of language abilities. Prior studies suggest that transcranial direct current stimulation (tDCS) directed toward language areas of the brain may help to ameliorate symptoms of PPA. In the present sham-controlled study, we examined whether tDCS could be used to enhance language abilities (e.g., picture naming) in individuals with PPA variants primarily characterized by difficulties with speech production (non-fluent and logopenic). Participants were recruited from the Penn Frontotemporal Dementia Center to receive 10 days of both real and sham tDCS (counter-balanced, full-crossover design; participants were naïve to stimulation condition). A battery of language tests was administered at baseline, immediately post-tDCS (real and sham), and 6 weeks and 12 weeks following stimulation. When we accounted for individuals’ baseline performance, our analyses demonstrated a stratification of tDCS effects. Individuals who performed worse at baseline showed tDCS-related improvements in global language performance, grammatical comprehension and semantic processing. Individuals who performed better at baseline showed a slight tDCS-related benefit on our speech repetition metric. Real tDCS may improve language performance in some individuals with PPA. Severity of deficits at baseline may be an important factor in predicting which patients will respond positively to language-targeted tDCS therapies.

Clinicaltrials.gov ID: NCT02928848

## Introduction

Primary Progressive Aphasia (PPA) is a neurodegenerative disorder characterized by gradual and initially isolated deterioration of language function (Mesulam, 2001). There are currently three recognized variants of PPA; semantic, non-fluent/agrammatic and logopenic. Semantic variant PPA (svPPA) involves anomia, reduction of expressive vocabulary and a severe single-word comprehension deficit, and involves atrophy of the anterior and ventral temporal lobe (Hodges and Patterson, 2007; Gorno-Tempini et al., 2011; Grossman, 2012). Non-fluent/agrammatic (nfvPPA) and logopenic variant PPA (lvPPA) are both characterized by more prominent difficulties with language production; naPPA typically involves grammatical simplification, effortful speech and motor speech impairment, and involves atrophy of the left inferior frontal lobe and insula (Ogar et al., 2007; Gorno-Tempini et al., 2011; Grossman, 2012), while individuals with lvPPA have trouble with word retrieval and repetition, and show atrophy of the left temporal and parietal lobes (Gorno-Tempini et al., 2011; Grossman, 2012). There are currently no effective treatments for PPA. Traditional speech and language therapies used in rehabilitation of post-stroke aphasia (e.g., Brady et al., 2012; Otal et al., 2015), have yielded limited benefits for PPA patients. However, recent research in the field of noninvasive brain stimulation shows promise for the development of symptom-oriented therapies (Wang et al., 2013).

Transcranial direct current stimulation (tDCS) is a type of noninvasive brain stimulation that modulates the resting excitability of neuronal populations, thereby altering patterns of brain activity in potentially behaviorally relevant ways (Stagg and Nitsche, 2011). The technique involves the application of low-intensity electrical current through electrodes placed on the scalp. A commonly invoked, but highly oversimplified, convention is that the application of anodal tDCS produces excitatory effects in underlying brain regions, and that cathodal stimulation is associated with inhibitory neural effects (Creutzfeldt et al., 1962; Nitsche and Paulus, 2000; Nitsche et al., 2008). However, some studies have highlighted that this traditional claim may not be entirely consistent depending on individual study parameters (Vallar and Bolognini, 2011; Batsikadze et al., 2013).

TDCS has been used to examine causal relationships between brain regions or networks and a variety of cognitive functions, including language processing (Nitsche and Paulus, 2000; Wiener et al., 2010; Turkeltaub et al., 2012; Chrysikou et al., 2013; Filmer et al., 2014; Price et al., 2015). A variety of language mechanisms have been interrogated with tDCS, such as word learning (Flöel et al., 2008; Fiori et al., 2011) and semantic verbal fluency (Cattaneo et al., 2011; Meinzer et al., 2012; Vannorsdall et al., 2012; Penolazzi et al., 2013). A recent meta-analysis of language processing in healthy adults found significant effects of single-session tDCS compared to sham across 11 studies (Price et al., 2015).

A number of left-hemispheric, anodal tDCS studies in patients suffering from post-stroke aphasia have shown promising effects of tDCS in language recovery (Flöel et al., 2008; Fridriksson et al., 2011; Cotelli et al., 2014; Wu et al., 2015). Anodal tDCS over the left frontal cortex of stroke patients with aphasia led to significant improvement in naming accuracy lasting 1 week following stimulation (Baker et al., 2010). However, therapeutic outcomes of tDCS studies across different studies are variable. Polanowska et al. (2013) found no statistically significant differences between anodal and sham tDCS over Broca’s area in naming accuracy or response time in post-stroke, non-fluent aphasic patients.

The use of tDCS in treating symptoms of neurodegenerative disorders has been studied to a lesser degree, with mixed findings for the efficacy of tDCS in these populations (see Elder and Taylor, 2014 for meta-analysis). Only a handful of studies have investigated the utility of tDCS for PPA symptoms specifically. Cotelli et al. (2014) found that 10 sessions of anodal tDCS over the left dorsolateral prefrontal cortex in combination with individualized speech therapy led to significant improvement in picture-naming (action and object naming) that lasted up to 12 weeks post-stimulation. However, the authors also reported significant performance gains in individuals who received only sham tDCS lasting the same amount of time, though these gains were smaller following sham relative to real tDCS. These results suggest that tDCS may enhance the outcome of intensive, targeted speech therapies, but do not indicate that tDCS on its own may be an effective intervention.

A recent case study of an individual with nfvPPA demonstrated improvements in auditory word-picture identification, picture naming, oral world reading and word repetition in the absence of speech therapy after 5 days of twice-daily anodal tDCS over the left posterior peri-Sylvian region (in the morning) and the left inferior frontal gyrus (in the afternoon; Wang et al., 2013). However, these improvements were modest and were not assessed at time-points following the conclusion of stimulation sessions.

Tsapkini et al. (2014) found that tDCS applied to the left inferior frontal gyrus paired with spelling therapy showed improvements in spelling lasting up to 2-months post-stimulation on untrained items compared to a sham control in six individuals with nfvPPA (*n* = 2) and lvPPA (*n* = 4). A double-blind, sham-controlled counterbalanced cross-over design study involving 12 patients with svPPA and 15 healthy subjects found that left-excitatory (anodal) and right-inhibitory (cathodal) tDCS to the temporal poles improved semantic accuracy in verbal modality among individuals with svPPA (Teichmann et al., 2016). Finally, a recent open-label study from our study team has demonstrated that 10 consecutive (5 weekdays for 2 weeks, with no stimulation on weekend days) sessions of anodal tDCS led to improvements in speech production, grammatical comprehension and semantic processing in patients with nfvPPA, some of which lasted up to 12 weeks post-stimulation (Gervits et al., 2016).

The main objective of the current study was to determine if tDCS, unpaired with individualized language therapy, can be used as a therapeutic tool to improve language impairments in patients with nfvPPA and lvPPA. We pursued this question using a blinded, sham-controlled crossover design in which participants were naïve to stimulation type and served as their own control. Additionally, we aimed to assess whether there are specific individual difference factors that may help to account for variability and possible tDCS-related improvements in language function in order to help determine whether and when tDCS may be appropriate to employ as a language therapy in PPA. One factor that we were specifically interested in exploring was baseline severity. Limited data from cohorts of healthy subjects suggest that performance on baseline assessment can be an important determinant of tDCS effects; individuals with weaker baseline performance have exhibited more consistent improvement than subjects with better baseline performance in several studies (Turkeltaub et al., 2012; Sarkar et al., 2014; Benwell et al., 2015). We hypothesized that real tDCS would be associated with improved language performance relative to sham, and that these improvements may be more or less pronounced depending on individual differences in baseline language performance.

## Materials and Methods

### Participants

Fifteen patients with a diagnosis of either nfvPPA or lvPPA were recruited from a large cohort of research participants at the Frontotemporal Degeneration Center at the University of Pennsylvania. All participants had been evaluated by a neurologist at the University of Pennsylvania and had received clinical diagnoses of PPA. Patients were excluded who were non-native English speakers, or who had a history of small vessel ischemic disease, seizures, other neurological conditions, unexplained loss of consciousness, or surgical breach of the skull. Patients who scored below 15 on the Mini-Mental State Exam (MMSE) were also excluded due to concern with global impairments precluding adequate comprehension and execution of task instructions. The study was approved by the Institutional Review Board at the University of Pennsylvania and all participants provided informed consent prior to participation.

**Figure 1:**
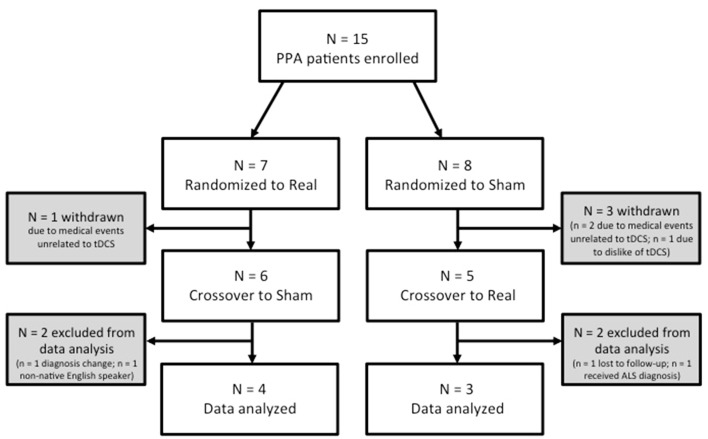
Recruitment and attrition information.

Of the 15 participants recruited, seven are included in the present analyses (Figure 1). Four participants withdrew prior to completing the protocol (two due to medical events unrelated to tDCS; one due to decline and unfeasibility of travel; one due to dislike of tDCS sensation). One participant was lost to follow-up prior to the final language assessment. Two participants received a change of diagnosis during or after completion of the study. Finally, one participant was excluded for being a non-native English speaker.

Our final sample for analysis consisted of five females and two males with a mean age of 68.71 years (Range = 58–79 years, *SD* = 6.97 years) and mean education of 13.86 years (Range = 10–18 years, *SD* = 2.73 years; see also Table 1). Our sample included patients with nfvPPA and lvPPA, although it was biased in favor of non-fluent/agrammatic PPA (six nfvPPA; one lvPPA)^1^. Patients reported varying time since the onset of their symptoms (*M* = 4.29 years, *SD* = 1.89 years). Four participants were randomized to receive real tDCS first and three received sham first.

**Table 1:** Demographic information.

# Males/Females	2/5
Age	68.71 ± 6.97
Years of education	13.86 ± 2.73
MMSE score at screening	24.40 ± 4.77
Diagnosis (IvPPA/nfPPA)	1/6
Disease duration at baseline (years)	4.29 ± 1.89
tDCS order (real first/sham first)	4/3

### Study Design

#### Overview

**Figure 2:**
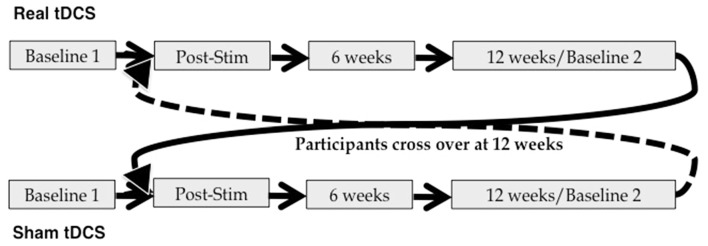
Study design. Participants were randomized to begin in either the real or sham arm of the study, and crossed over at 12 weeks post-stimulation.

This was a blinded, randomized, sham-controlled tDCS study. Subjects received 10 daily sessions of real or sham tDCS (Monday–Friday × 2 weeks), employing the stimulation parameters detailed below. Neuropsychological evaluation was administered at baseline (T0) and immediately following the final stimulation session (T1). Follow-up assessments were conducted at 6 weeks (T2) and 12 weeks (T3) post-stimulation. The T3 assessment also served as a second baseline measure for participants as they crossed over into the next arm of the study. This was done for two reasons: first, it allows for examination of the time-course of any tDCS effects in arm 1; and second, it allows us to account for possible carry-over effects of stimulation in examining performance during and following tDCS in arm 2. Immediately after the T3 assessment, participants began a second 10-day round of tDCS. If they had received real stimulation first, they crossed over into the sham condition; if they received sham first, they crossed over into the real condition. Additional assessments were administered immediately post-stimulation (T4), as well as 6 weeks (T5) and 12 weeks (T6) post-stimulation (see also Figure 2).

#### tDCS Procedures

**Figure 3:**
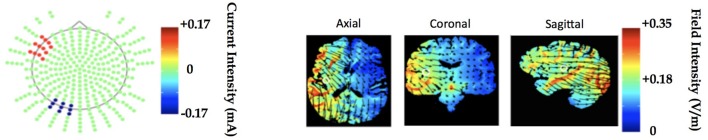
Electrode montage and underlying left hemisphere cortical regions to be stimulated modeled in Soterix transcranialdirect current stimulation (tDCS)-Explore™. The model demonstrates current delivery to the F7 and O1 locations results in field intensity increase in regions associated with language processing.

tDCS was administered using a battery-driven Magstim Eldith machine. 5 × 5 cm electrodes were placed in saline-soaked pads and secured to the scalp with a rubber headband. Stimulation was delivered at 1.5 mA (current density = 0.06 mA/cm^2^) over a period of 20 min per session, with additional 30-s ramp-up and ramp-down periods at the start and end of stimulation, respectively. The anode was placed over the left prefrontal region (F7 in the International EEG 10–20 system; Homan et al., 1987), and the cathode was placed over the left occipital region (O1). This montage was identical to that used in Gervits et al. (2016). Since it is not possible to focally target specific brain regions with tDCS, this montage was selected for its capacity to influence activity broadly within the left hemisphere in order to target the left-lateralized language network (see Figure 3 for a theoretical model of current distribution in the brain associated with this electrode montage). Sham stimulation was delivered for 30 s with built-in ramp-up and ramp-down periods proportional to the total stimulation time, in this case approximately 11 s.

Because we were interested in whether tDCS can be used to improve speech production in PPA and to control the activity performed during stimulation across patients, we employed an unstructured language task during stimulation in which patients were asked to verbally narrate wordless children’s books during each stimulation session (real and sham). This task was not intended to serve as a therapeutic intervention in and of itself, simply to engage the language network during tDCS. Evidence indicates that cognitive activities pursued during stimulation can strongly influence the kinds of performance changes induced by stimulation (Andrews et al., 2011; Gill et al., 2015). A different book was used in each session and participants engaged in unstructured narration throughout each 20-min period of real or sham stimulation. Sessions were recorded to allow for the possibility of exploratory offline scoring, though we have no specific hypotheses regarding changes in narration ability during stimulation in the real or sham conditions.

#### Language Battery

A battery of linguistic assessments designed to evaluate a wide range of language abilities was administered to each participant by testers trained in the administration of psychometric assessments (FG, NW)^2^. All sessions were digitally audio-recorded for offline analysis by a coder blinded to time-point and tDCS type. For full detail regarding the language battery, see Gervits et al. (2016).

#### Outcome Measures

The tests employed in our language battery assess many domains of language performance, some of which are more or less severely affected in patients with nfvPPA and lvPPA. We created three composite measures that reflected common clinical features of these PPA variants. Speech repetition was assessed via performance on the Sentence Repetition test. Grammatical comprehension was assessed via performance on the Penn-TROG (Charles et al., 2014). Semantic processing was assessed via composite performance across the BNT, PPT and Category Fluency tests. Finally, scores across all tests within the language battery^3^ were combined into one composite measure to facilitate assessment of overall language performance across domains (Global Performance). Table 2 shows the distribution of performance across participants at T0.

**Table 2:** Spread of low and high performers across tasks at baseline.

		Grammatical comprehension	Semantic processing			Speech repetition
Subject	First arm	Penn-TROG (out of 36)	BNT (out of 15)	Category fluency (no ceiling)	PPT (words and pictures: out of 52)	Sentence repetition (out of 5)
DM017	Real	High	High	High	High	Low
GM016	Real	High	Low	High	High	High
KC012	Real	Low	Low	Low	Low	Low
UG015	Real	High	High	High	High	High
EH021	Sham	High	High	High	High	High
KC014	Sham	Low	Low	Low	Low	Low
TN009	Sham	Low	High	Low	Low	High
	Low performer mean	21.33	5.33	10.67	38.00	0.67
	Low performer SD	2.31	4.04	4.04	2.65	1.15
	High performer mean	28.25	14.00	21.50	48.75	4.00
	High performer SD	3.20	1.41	11.90	3.40	0.82

### Data Analysis

Scores on each test within the language battery were separately converted to z-scores based on the mean and standard deviation across all participants and time-points (T0–T6). These transformations facilitated comparisons of performance following tDCS across tests with different scoring metrics and different numbers of items (e.g., the BNT has 15 items, while the Penn-TROG has 36 items). Where scores from multiple tests were combined into composites, data were rescaled such that z-score differences would be considered under one distribution. Difference scores were computed for each time-point relative to the most recent baseline (T1-T0; T2-T0; T3-T0; T4-T3; T5-T3; T6-T3) in order to assess the magnitude of change from baseline as measured in units of standard deviation. Thus, for the first arm of tDCS, we used T0 as the baseline measure for computing difference scores for T1 through T3; for the second arm of tDCS, we used T3 as the baseline for computing difference scores for T4 through T6. This was done to account for any possible order effects regarding the administration of tDCS.

**Figure 4:**
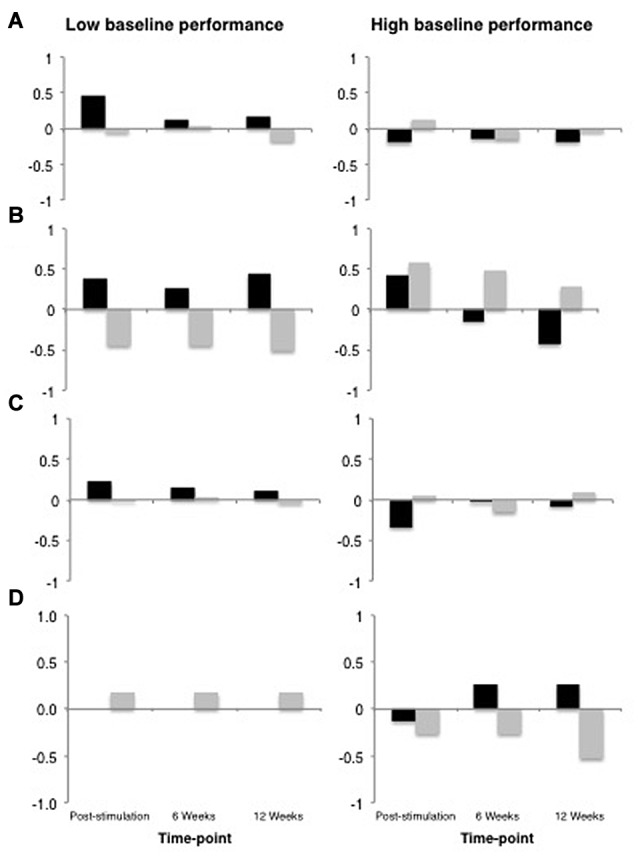
Data from across all time-points plotted for descriptive purposes. In all panels, real tDCS outcomes are depicted in black and sham outcomes are depicted in gray, and the *y*-axis represents z-score change from the most recent baseline. **(A)** Global Performance. **(B)** Grammatical Comprehension. **(C)** Semantic Processing. **(D)** Speech Repetition.

**Figure 5:**
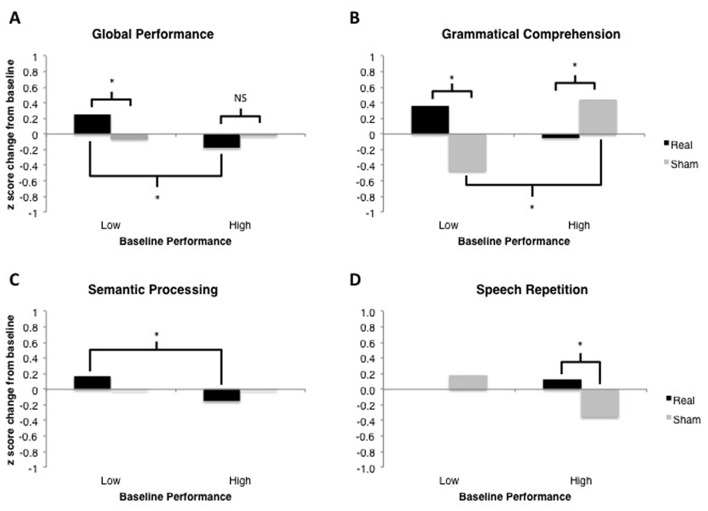
Results of linear mixed-effects tDCS × Baseline Performance analyses for each language domain of interest. Model-estimated means are plotted in units of z-scores measured as change relative to the most recent baseline (i.e., standardized different scores). Asterisks represent significant comparisons at the *p* < 0.05 level. **(A)** Global Performance of low and high performers at baseline. **(B)** Grammatical Comprehension of low and high performers at baseline. **(C)** Semantic Processing of low and high performers at baseline. **(D)** Speech Repetition of low and high performers at baseline.

**Figure 6:**
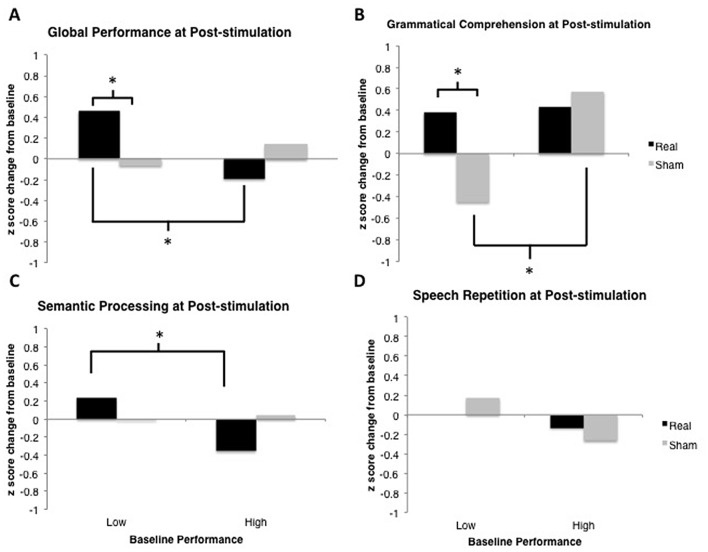
Results from the post-stimulation time-point only. Model-estimated means are plotted in units of z-scores measured as change relative to the most recent baseline (i.e., standardized different scores). Asterisks represent significant comparisons at the *p* < 0.05 level. **(A)** Global Performance at post-stimulation time-point. **(B)** Grammatical Comprehension at post-stimulation time-point. **(C)** Semantic Processing at post-stimulation time-point. **(D)** Speech Repetition at post-stimulation time-point.

All data analyses were performed using R (R Core Team, 2016), and the R packages *lme4* v1.1-9 (Bates et al., 2015), *languageR* v1.4.1 (Baayen, 2013) and *LMERConvenienceFunctions* v2.10 (Tremblay and Ransijn, 2015) using multilevel modeling with maximum-likelihood estimation (Faraway, 2006; Baayen et al., 2008). For each outcome measure, we performed linear mixed-effects modeling analyses to examine: (1) the effect of tDCS Type (real vs. sham) as a sole predictor of performance; and (2) the possible interactive effects of tDCS Type × Baseline Performance (median split) on performance. In the present set of analyses, we did not have specific *a priori* predictions about the time-course of possible tDCS-related benefits nor sufficient power to detect any potential three-way interaction between tDCS, time-point and baseline performance. Figure 4 shows the data across all time-points for descriptive purposes. For the most part, the general pattern of outcomes shows the largest change immediately following stimulation and decaying over time. An additional analysis of each of our outcome variables restricted to the post-stimulation time-point only revealed no substantial differences in statistical findings (with the exception of Speech Repetition^4^; see “Results” Section and Figures 5, 6), thus we have opted to present data collapsed across time-points both due to enhanced power as well as a potentially more stable, conservative estimate of the effects of tDCS without specific time-course predictions.

## Results

### Safety and Tolerability

Generally, participants reported experiencing mild itching during the initial period of stimulation that declined after the first few minutes. One participant experienced slight skin irritation during both real and sham stimulation under the F7 electrode that dissipated after stimulation ended. As mentioned above, one participant withdrew from the study on the third day of sham stimulation due to dislike of the sensation associated with stimulation. There were no other adverse effects reported during either real or sham stimulation.

### Baseline Performance

Participants were categorized as low performers or high performers based on their language outcomes at T0. Because we are using a linear mixed-effects approach, we chose to classify performance on each language task individually, to better account for individual variability in performance. For example, a patient with nfvPPA may be relatively more impaired on tasks that require speech production as compared to tasks that can be completed without speech. Rather than assigning a composite “average” level of performance to each participant (e.g., for our Global Performance metric), which may obscure possible across-task variance within a subject, we used a median split procedure for each task and retained this level of resolution in our linear mixed-effects analyses. Table 2 shows the division of participants into low- and high-performing categories for the tasks comprising the composite measures we present here, as well as the tDCS condition in which each individual began the study.

### Assessment of Model Viability

Due to our small sample size, we examined the residuals of each of the four interaction models presented below to ensure that our data did not violate the assumption of normally distributed model residuals. Table 3 provides estimates of the mean, median and skewness for the residuals of all models, each discussed in more detail below.

**Table 3:** Model residuals for each domain of analysis.

Domain	Mean	Median	Skewness^a^	Shape
Global performance	−2.16E-12	0.0012	0.756	Unimodal
Grammatical comprehension	7.14E-11	0.0856	−0.278	Bimodal*
Semantic processing	−3.57E-11	−0.0239	0.401	Unimodal
Speech repetition	−9.52E-11	0	−1.062*	Unimodal

*Asterisks indicate a violation of the expected normal distribution. ^a^Data simulations support a skewness cutoff of ±0.963 for a study with *n* = 10 sample size (see Doane and Seward, 2011 for detail). Generally speaking, skewness cutoffs scale in inverse proportion to sample size, thus we conservatively use this *n* = 10 cutoff for our evaluations*.

### Global Performance

#### Effect of tDCS Type

Linear mixed-effects modeling revealed no main effect of tDCS Type on global performance change from baseline, *F*_(1,453)_ < 1.

#### tDCS Type × Baseline Performance

This analysis demonstrated no significant main effect of tDCS Type, *F*_(1,451)_ < 1. There was a marginally significant main effect of Baseline Performance, *F*_(1,451)_ = 3.37, *p* = 0.067. The two-way tDCS Type × Baseline Performance interaction was significant, *F*_(1,451)_ = 6.76, *p* = 0.0096 (see Figure 5A). Examination of the fixed effects structure in the model revealed that individuals who scored lower at baseline improved significantly following real tDCS (*M* = 0.255) relative to their own sham tDCS (*M* = −0.062), *t*_(452.2)_ = −2.491, *p* = 0.013. There was no such difference in performance following real vs. sham tDCS for participants who scored high at baseline, *t*_(452.2)_ = 1.19, *p* = 0.233. Additionally, performance change following real tDCS was significantly greater for low baseline scorers (*M* = 0.255) relative to high baseline scorers (*M* = −0.176), *t*_(367.3)_ = 3.08, *p* = 0.0022. There was no difference in performance change between low and high baseline scorers following sham tDCS (*Ms* = −0.062 and −0.022, respectively), *t*_(367.3)_ = −0.281, *p* = 0.779.

### Grammatical Comprehension

#### Effect of tDCS Type

Linear mixed-effects modeling revealed no main effect of tDCS Type on grammatical comprehension change from baseline, *F*_(1,33)_ < 1.

#### tDCS Type × Baseline Performance

This analysis demonstrated no significant main effect of tDCS Type, *F*_(1,31)_ < 1. There was also no main effect of Baseline Performance, *F*_(1,31)_ = 2.22, *p* = 0.146. The two-way tDCS Type × Baseline Performance interaction was significant, *F*_(1,31)_ = 4.56, *p* = 0.0005 (see Figure 5B). Examination of the fixed effects structure in the model revealed that individuals who scored lower at baseline improved significantly following real tDCS (*M* = 0.364) relative to sham tDCS (*M* = −0.471), *t*_(38)_ = −3.24, *p* = 0.003. Conversely, for participants who scored high at baseline, performance improved significantly following sham (*M* = 0.449) compared to real tDCS (*M* = −0.048), *t*_(38)_ = 2.23, *p* = 0.032. Following sham tDCS, low baseline scorers (*M* = −0.471) improved significantly less than high baseline scorers (*M* = 0.449), *t*_(38)_ = −3.82, *p* = 0.0004. There was no difference in performance change between low and high baseline scorers following real tDCS (*Ms* = 0.364 and −0.048, respectively), *t*_(38)_ = 1.71, *p* = 0.096. However, given the bimodal distribution of model residuals (see Table 3), these results must be interpreted with caution. Bimodal model residuals suggest some systematicity to prediction error in the model that may reflect a non-linear relationship between grammatical comprehension performance and PPA severity. However, it is also possible that this finding is related only to the size of the dataset, and that model residuals would approach normality with an increased sample size. More data are needed to clarify this finding.

### Semantic Processing

#### Effect of tDCS Type

Linear mixed-effects modeling revealed no main effect of tDCS Type on semantic processing change from baseline, *F*_(1,159)_ < 1.

#### tDCS Type × Baseline Performance

This analysis demonstrated no significant main effect of tDCS Type, *F*_(1,157)_ < 1, or of Baseline Performance, *F*_(1,157)_ < 1. The two-way tDCS Type × Baseline Performance interaction was marginally significant, *F*_(1,157)_ = 3.38, *p* = 0.068 (see Figure 5C). Examination of the fixed effects structure in the model showed that performance change for individuals who scored low at baseline (*M* = 0.164) improved significantly following real tDCS relative to those who scored high at baseline (*M* = −0.152), *t*_(130.4)_ = 2.12, *p* = 0.036. No other comparisons were significant (all *p*s > 0.15).

### Speech Repetition

#### Effect of tDCS Type

Linear mixed-effects modeling revealed no main effect of tDCS Type on speech repetition change from baseline, *F*_(1,33)_ = 1.91, *p* = 0.176.

#### tDCS Type × Baseline Performance

This analysis demonstrated no significant main effect of tDCS Type, *F*_(1,31)_ = 2.17, *p* = 0.150, and no main effect of Baseline Performance, *F*_(1,31)_ < 1. The two-way tDCS Type × Baseline Performance interaction was significant, *F*_(1,31)_ = 5.73, *p* = 0.023 (see Figure 5D). Evaluation of model residuals revealed only a slight deviation from normality according to our skewness cutoff at the *p* = 0.05 level (−1.062 vs. 0.963, respectively; see Doane and Seward, 2011). However, we note that we have employed a conservative skewness cutoff (based on sample size of *n* = 10 rather than *n* = 7) in making this determination. Examination of the fixed effects structure in the model revealed that individuals who scored higher at baseline improved significantly following real tDCS (*M* = 0.132) relative to sham tDCS (*M* = −0.353), *t*_(33)_ = −2.68, *p* = 0.011. There was no such difference in performance following real vs. sham tDCS for participants who scored low at baseline, *t*_(33)_ = 0.85, *p* = 0.404. No other comparisons were significant.

## Discussion

The present study employed a randomized, sham-controlled design to assess the potential of tDCS as a therapy to modulate language difficulties in patients with PPA. Whereas a previous open-label study demonstrated large effects across all domains assessed (see Gervits et al., 2016), the same comparisons made with our sham-controlled design revealed no significant findings in any domain. However, when we took into account each individual’s language performance at the baseline assessment (T0), we were able to demonstrate the importance of baseline performance in predicting which patients will respond positively to tDCS, as indexed by an improvement in language performance. Generally speaking, individuals whose performance was lower at baseline demonstrated greater propensity to improve after receiving real tDCS relative to sham tDCS. This was the case for our metric of Global Performance. We also observed this pattern of results for Grammatical Comprehension performance, though there was observable bimodality in the residuals of this model that must be taken into account when interpreting the outcome of the present analysis. Individuals whose performance was lower at baseline also demonstrated significant improvement in Semantic Processing following real tDCS compared to individuals who performed better at baseline, although this improvement was not significant relative to the sham condition.

The only measure in which higher performance at baseline was associated with tDCS-specific outcomes was in our Speech Repetition test (Figure 4D). However, the significant difference in performance between real and sham conditions appeared to reflect a *decline* in performance following sham tDCS rather than a tDCS-related improvement. Relative to baseline performance, there was no significant improvement following real tDCS. It is difficult to interpret this finding given the lack of statistically significant improvement following real tDCS relative to baseline. It is possible that this pattern of results is due to a “protective” effect of real tDCS, such that the application of tDCS may prolong the maintenance of speech repetition in the course of the disorder. That is, individuals who started out with better performance may have experienced greater decline in speech production abilities over the course of the 6 months of the study, possibly related to selective disease progression, whereas individuals whose speech was already affected may have shown more stable error rates. Anecdotally, individuals who made many errors in speech repetition tended to make the same errors consistently, which may be reflected in stable change scores. However, another caveat to this interpretation is the nature of the Speech Repetition task itself, which comprises a total of five items. Given the small range across which to assess performance, it may be that low performers demonstrate a floor effect, such that any potential decline in ability cannot adequately be detected with this task. Further investigation is required to clarify the nature of this finding.

Given variability in the outcomes across tDCS studies, it is particularly important to examine potential modulating factors of individual response to tDCS. Participants who scored lower at baseline demonstrated greater tDCS-related benefits overall, suggesting (perhaps counter-intuitively) that tDCS may be more beneficial for patients who are treated at a later stage in the course of their disease. These findings are consistent with previous brain stimulation studies in which baseline performance was measured as a potentially influential factor on results. Benwell et al. (2015) found that bi-parietal left anodal/right cathodal tDCS effects were relative to a participant’s baseline performance on a perceptual line bisection task. In a cohort of cognitively healthy individuals, Turkeltaub et al. (2012) observed that tDCS-induced enhancement of reading efficiency was most consistently among subjects who had weaker reading efficacy at baseline. Moreover, Sarkar et al. (2014) found that otherwise healthy subjects who had high math anxiety (a predictor of poorer mathematical performance) temporarily benefitted from a mathematical (arithmetic) training task paired with tDCS, while participants who had low baseline math anxiety (and presumably higher math ability) got transiently worse as a result of receiving tDCS. A similar study also found that greater cognitive gains were achieved by individuals with lower baseline performance on a mathematical video game when paired with anodal tDCS (Looi et al., 2016).

Assessing baseline performance in patients with neurodegenerative disorders who are slated to receive tDCS may be especially important due to the theoretical mechanism of tDCS in influencing neuronal function. Because tDCS is thought to alter resting excitability of populations of neurons (Stagg and Nitsche, 2011), the degree of atrophy (likely related to the severity of symptoms at baseline) in affected regions may be a critical factor in deciding to whom tDCS should be prescribed and when. The current results suggest that application of tDCS in PPA patients whose symptoms are too mild may not be beneficial. On the other hand, if progression is too far along, it is also possible that tDCS intervention would be unhelpful due to advanced tissue loss in brain regions necessary for language function. Future work should further investigate the possible inverted-U “critical period” for tDCS intervention in PPA. Though we did not assess baseline cortical thickness in the present study, future exploration of the influence of baseline symptom severity should take into account the progression of cortical atrophy as a possible predictor of response to tDCS.

Participants who score higher on tests of language performance at the baseline assessment may not benefit as much from tDCS due to the mildness of deficits. Since our analyses focused on *change* in performance rather than overall performance, higher performing participants may have delivered more stable performances across time points, leaving less room for the tDCS intervention to have an effect. On the other hand, participants who scored lower at baseline may have had more room for improvement, and thus exhibited greater performance gains following tDCS. Previous studies (e.g., Cotelli et al., 2014) have purposely enrolled patients with mild language deficits, but have paired tDCS with intensive, targeted language therapies. Combining therapies in this way may help improve symptoms in individuals with milder deficits, whereas tDCS alone may provide some benefit in individuals whose symptoms have progressed further. The degree to which combination speech therapy-tDCS interventions may help with more severe PPA symptoms is currently unknown. A caveat of this explanation is that our metrics may not have been sensitive enough to detect performance change in participants who were high-performing at baseline. Since all individuals enrolled were experiencing language-related difficulties at the time of study, it is possible that evaluating language performance in other ways (e.g., via metrics that combine performance accuracy and speed to assess language *efficiency* rather than absolute test scores) may be more sensitive to the possible tDCS-related enhancement of performance in individuals whose symptoms are less severe. Elucidating the capacity of tDCS to remediate symptoms with and without concurrent speech therapy at different stages of PPA progression will be critical to determining the application of tDCS as a therapy for people with PPA.

We did not expect to find such a dramatic difference in the outcome of the present study as compared to Gervits et al. (2016). Whereas the previous open-label sample demonstrated significant language gains across all domains tested, similar analyses on the current dataset revealed no significant tDCS-related improvements until baseline performance was taken into account. A few key factors may explain these differences. First, there was an unequal representation of lvPPA and nfvPPA subjects in each study. The prior study included four individuals with lvPPA and two with nfvPPA, while the current study included only one lvPPA patient and six nfvPPA patients. Though both of these PPA variants involve difficulties biased toward language production (as compared to comprehension), the symptomatology of these two neurodegenerative diseases is expressed differently and may explain varied outcomes on language measures following tDCS. Since a key characteristic of nfvPPA is difficulty with grammatical comprehension and repetition (Gorno-Tempini et al., 2011), this may have had an effect on performance of these language measures. Similarly, we encourage future studies to develop more granular* a priori* hypotheses regarding which language abilities could be affected by the inclusion of a more specific task paired with stimulation. This additional specification would allow for stronger inferences to be made regarding the effects of stimulation within the language network. Finally, the findings across our two studies emphasize the importance of cautious interpretation in the setting of a potential placebo effect, and the critical role of a sham control condition. Future studies should delineate further distinctions between the variants of PPA and the associated improvements or lack thereof across different language measures.

### Limitations of the Current Study

One limitation of our study is the small sample size and skewed distribution of PPA variants. We do not have a large enough sample to assess whether nfvPPA or lvPPA patients are relatively more likely to benefit from tDCS, or whether this may be true to different extents across different domains of language.

Additionally, the natural time-course of language decline in PPA is not well understood. This is of particular relevance in determining whether tDCS is a useful therapy for PPA patients, since the degree to which *improvement* and *lack of decline* may both be reflective of a positive tDCS outcome. In the latter instance, it may be the case that early tDCS intervention delays decline in individuals who are higher-performing at baseline, but we cannot currently distinguish such an outcome from a null effect.

The current study did not aim to develop specific hypotheses regarding the interaction between performing a task and tDCS. We can infer that the act of narrating wordless picture stories requires engagement of the language network, specifically object recognition, semantic processing, verbal working memory, grammatical processing and phonological processing among other language functions. This unstructured task was employed to broadly enhance language production during stimulation, however no further predictions were made regarding this interaction.

Finally, in the current study we did not have enough power to examine the time-course tDCS-related language benefits to determine how long improvement lasts, which may also be affected by baseline performance and will be important in assessing the therapeutic value of tDCS intervention.

## Conclusion

The current results suggest that language abilities at baseline are a strong predictor of tDCS-mediated symptom management in individuals with PPA. Further research is needed to clarify the role of tDCS at different stages of this progressive disorder, specifically to assess whether tDCS may be more effective in treating symptoms in specific PPA variants, and when to begin therapy.

## Author Contributions

RHH, HBC, MG and SA designed the experiments; FG, NCW and EMM performed the experiments and designed analysis methods; NCW analyzed the data; and EMM, NCW and RHH wrote the manuscript.

## Conflict of Interest Statement

The authors declare that the research was conducted in the absence of any commercial or financial relationships that could be construed as a potential conflict of interest.

## Footnotes

^1^Given the imbalance in diagnosis of participants included in this sample, we visually examined individual subjects’ raw scores across all metrics within the language battery. There were no apparent systematic differences in the pattern of outcomes for lvPPA vs. nfvPPA. To confirm this statistically, an exploratory analysis of Global Performance excluding this participant showed no change in statistical outcomes. Therefore, we have chosen not to exclude our lvPPA patient from these analyses.

^2^It was not possible to ensure that a single tester administered all assessments. To account for the potential confounding factor of test administrator, we included this variable as a covariate in a linear mixed-effects modeling analysis of global performance on the battery and found no significant effect of test administrator on performance.

^3^See Gervits et al. (2016) for full details. We elected to leave the Speech Production metric out of the current analyses. This composite score is computed from several aspects measured in spontaneous speech production during the Cookie Theft Picture Narrative task. Analysis of this rich data set is beyond the scope of this analysis, and will be addressed a separate future manuscript.

^4^Speech Repetition was the only domain for which a difference in statistical outcomes was observed when comparing results at the post-stimulation time-point to results across all time-points (see Figures 5, 6). Specifically, the reduction in Speech Repetition scores following sham tDCS for participants who scored high at baseline was not significant at the post-stimulation time-point, indicating that the decline in scores seen in the overall analysis is likely driven by change at the 6- and/or 12-week time-points. It is difficult to draw conclusions regarding this outcome without appealing to interpretation of null findings in the real tDCS condition, but we suggest that this decline over later time-points may reflect progression of symptoms in high performers, and address this further in the “Discussion” Section.
